# Isolation and antimicrobial drug susceptibility pattern of bacterial pathogens from pediatric patients with otitis media in selected health institutions, Addis Ababa, Ethiopia: a prospective cross-sectional study

**DOI:** 10.1186/s12901-018-0056-1

**Published:** 2018-05-24

**Authors:** Tewodros Tesfa Hailegiyorgis, Wondemagegn Demsiss Sarhie, Hailemariam Mekonnen Workie

**Affiliations:** 1 Department of Medical Laboratory Science, College of Medical and Health Sciences, Haramaya University, P.O.BOX, 235 Harar, Ethiopia; 2 Department of Medical Laboratory Sciences, College of Medicine and Health Sciences, Wello University, P.O.BOX, =1145 Dessie, Ethiopia; 3 School of Nursing and Midwifery, Department of Pediatric Nursing, College of Health and Medical Sciences, Haramaya University, P.O.BOX, 235 Harar, Ethiopia

**Keywords:** Otitis media, Bacterial pathogens, Antimicrobial susceptibility, Pediatric patients

## Abstract

**Background:**

Otitis media is inflammation of the middle ear and tympanic membrane, which often occurs after an acute upper respiratory tract infection. It is the most common episode of infection in children and the second most important cause of hearing loss affecting 1.23 billion people, thus ranked fifth global burden of disease with a higher incidence in sub-Saharan Africa. Thus, the aim of this study was to determine the isolation rate of bacterial pathogens from pediatric patients with otitis media.

**Methodology:**

Institutional based cross-sectional study was conducted from January 2013–June 2014 in Addis Ababa among 210 pediatrics patients. Demographic, clinical and associated factors data was obtained in face to face interview with guardians/parents by 5 trained nurse data collectors using structured questionnaire. Middle ear drainage swab was collected following all aseptic procedures and transported to the microbiology laboratory. Culture and Antimicrobial sensitivity test were performed according to the standards. The data quality was assured by questionnaire translation, retranslation and pretesting. Reference strains were used as a positive and negative control for biochemical tests, and culture results were cross checked. Data was checked for completeness, consistency and then entered into Epi Info v3.5.1 and analyzed by SPSS v20. Data interpretation was made using graphs, tables, and result statements.

**Result:**

A total of 196 middle ear drainage swab samples were analyzed from pediatric patients and of those 95 (48.5%) samples were positive for pathogenic organisms. The major isolate was *S. aureus* (15.8%) followed by *P. aeruginosa* (10.9%), Viridians streptococcus (9.9%), *S. pneumoniae* (8.9%) and *S. pyogenes* (7.9%). Upper respiratory tract infection history and living in the rural area have shown significant association with the isolation of pathogenic organism, (*p-value = 0.035)* and (*p-value = 0.003)* respectively. Most of the isolates show a high level of resistance to Trimethoprim-Sulfamethoxazole, Penicillin G, Ampicillin, Amoxicillin, and Chloramphenicol.

**Conclusion:**

*S. aureus* and *P. aeruginosa* are the most common pathogens that contribute to otitis media as well most of the isolates show a high level of resistance to commonly used drugs to treat otitis media. Therefore, culture and susceptibility testes have paramount importance for the better management of otitis media and drug-resistant infections.

## Background

Otitis media is an inflammation of the middle ear and the tympanic membrane, mostly after an acute upper respiratory tract infection [1]. Otitis media is the most common episode of infection in children and the second most important cause of hearing loss affecting 1.23 billion people, thus ranked fifth global burden of disease [2, 3]. It is one of the major chronic disease in low and middle-income countries and the most common reason for which children receive antibiotics [2–4]. Otitis media have 2–8 fold higher incidence in Sub-Saharan Africa and South Asia than developed world [4, 5] and the associated complications lead to the death of around 20,000 people annually, the highest mortality rate being in < 5 children [5]. Hearing loss, reduced learning ability, and low scholastic achievement have been indicated due to the chronic and recurrent form of the disease [6]. The major pathogens being bacteria like non typeable *H. influenzae, S. pneumoniae, S. aureus, S. pyogenes, P. aeruginosa, P. mirabilis, E. coli* and *M. catarrhalis* [7–11], otitis media can also be caused by virus and fungi [10, 12]. Viral upper respiratory tract infections (URTI) predispose children to acute otitis media (AOM) by disrupting the mucociliary system, impairing the host’s primary mechanical defense from bacterial invasion, and viruses alone can cause AOM [8, 11]. An episode of otitis media is directly associated to the bacterial colonization rate of the nasopharynx [8]. In Ethiopia, few studies have indicated that there is a high level of antimicrobial resistance isolates from otitis media [13, 14]. The true picture of otitis media in under five-year-old children is not known in Addis Ababa and many general practitioners and pediatricians base their treatment on empirical evidence of the etiologic agent and susceptibility to commonly used antibacterial drugs. Knowing the local antibiogram is important for cost-effective and appropriate treatment of otitis media and help prevent its complications that may arise due to the luck of treatment or improper treatment. Thus, the aim of this study was to acquire data on bacterial pathogens responsible for otitis media and their antibacterial susceptibility pattern among under five children who come to visit public health institutions in Addis Ababa.

## Methods

### Study area and period

The study was conducted from January 2013 to June 2014 in five selected public health institutions namely: Yekatit 12 Medical College Hospital, Kebena Health center, Shero-meda Health center, Arada Health center and Teklehaimanot Health center at Addis Ababa Ethiopia.

### Study design and population characteristics

Institutional based cross-sectional study was conducted in a total of 210 pediatric patients under the age of 5 years. Participant children were selected conveniently based on their clinical presentations. Pediatrics patients who had started antibiotics within the past two weeks from the sample collection day and children with otitis externa were excluded from the study.

### Data collection and sample processing

Demographic, clinical and risk factors data was obtained in face to face interview with guardians/parents by 5 trained nurse data collectors using structured questionnaire. Middle ear drainage swab was collected by 1 ENT specialist and 4 trained nurses for microbiological analysis. Sample collection site for laboratory test was cleaned with sterile saline water and gauze to remove excess debris. The collected sample was transported by Amies transport medium (Oxoid, UK) to Yekatit 12 Medical college microbiology laboratory for microbiological analysis.

Specimens were inoculated directly on Blood agar, Chocolate agar and MacConkey agar (Oxoid, Basingstoke, and Hampshire, UK, England) following the standard procedure of inoculating culture media. Pathogens from positive culture results were identified by their characteristic appearance on the respective media, gram-staining reaction and pattern of biochemical profiles using standard procedures.

All isolated pathogens were tested for antibiotic susceptibility by Kirby - Bauer disc diffusion method. Inoculums were prepared by transferring 5 colonies from pure culture into 5 ml Nutrient Broth (Oxoid, England) and incubated at 35 °C for 2 h. Turbidity equivalent to 0.5 McFarland was obtained by diluting the broth and the entire surface of Muller Hinton agar and Muller Hinton agar supplemented with 5% defibrinated blood (for *M. catarrhalis* and Streptococcus species) was streaked with the inoculums using a cotton swab according to National Committee for Clinical Laboratory Standards [15] and British Society of Antimicrobial Chemotherapy [16]. The growth inhibition zone was measured by caliper and result was interpreted as weather the organism was sensitive, intermediate or resistant to the antimicrobial agents by comparing with standard guideline [15, 16].

### Data quality assurance

The data quality was assured by using different methods. Standard and structured questionnaire was used. The questionnaire was prepared in English and translated to the local language (Amharic) for data collection and then re translated back into English for analysis. 2 days training was given to the data collectors and supervisors on the data collection tool and procedures. Then the questionnaire was pretested on 5% of the sample size to ensure its validity. Findings from the pretesting were utilized for modifying and adjustment of the instrument and interviewing technique. Data collectors were supervised closely by the supervisors and the principal investigators. Completeness of each questionnaire was checked by the principal investigator and the supervisors on daylily basis. Double data entry was done by two data clerks and consistency of the entered data was cross checked by comparing the two separately entered data.

Middle ear drainage samples which need delicate care for patients were collected by ENT specialist and trained nurses. The quality of sample was checked by gram staining technique before proceeding to culture. Culture media were cheeked by culturing reference strains and also the media were incubated for overnight to make sure whether any contamination has occurred during culture media preparation. *Staphylococcus aureus* (ATCC 24923*)* and *Neisseria gonorrhoeae* (ATCC 43069) were used to control the functionality of Blood agar and Chocolate agar, whereas *Pseudomonas aeruginosa* (ATCC 27853) and *Escherichia coli* (ATCC 25922) were used to check the quality of MacConkey agar and KIA agar. In addition, reference strains were used as a positive and negative control for biochemical tests and results were cross checked by microbiologists at Yekatit 12 medical college microbiology laboratory.

### Statistical analysis

The collected data was checked for its completeness and cleaned before entry. Then the questionnaire was coded and entered into Epi Info version 3.5.1 by two data clerks. Then the data was exported to SPSS version 20 for further data cleaning and for analysis. Frequency was run to check for any missing values and checked accordingly. Descriptive statistics were used to present the prevalence of bacterial pathogens and Binary logistic regression was used to assess the association between the variables. Variables that yielded a *p*-value of < 0.25 in bivariate analysis was considered for multivariable logistic regression analysis to control all possible confounders and to detect true predictors.

For measuring the strength of the association between the outcome and independent variables, Crude Odd Ratio (COR) and Adjusted Odd Ratio (AOR) along with 95% Confidence interval (CI) was calculated and accordingly statistical significance was declared at *p*-value < 0.05.

## Results

### Study participants characteristics

A total of 210 middle ear swab samples from pediatric patients were collected during the study period. Out of 210 samples, 14 samples were rejected due to inappropriate collection and wrong sample, and the rest 196 samples were analyzed. The age of children ranged from 1 month of age to 5 years with a mean age of 2 years and 2 months (*SD = 1.578*) and a median age of 2 years (Table 1). The number of female pediatric patients (*51.5%*) was relatively higher than male pediatric patients (*48.5%*) and 86.73% of the children permanently live in urban area.

**Table 1:** Age and sex distribution of 196 pediatric patients investigated for otitis media in Addis Ababa, Ethiopia, Jan 2013- Jun 2014 (*n* = 196)

Age group	Male No.(%)	Female No.(%)	Total No.(%)
< 2 years	41 (49.4)	42 (50.6)	83 (42.35)
2–4 years	36 (48.65)	38 (51.35)	74 (37.76)
> 4–5 years	18 (46.15)	21 (53.85)	39 (19.9)
Total	95 (48.5)	101 (51.5)	196 (100)

### Prevalence of bacterial isolates

**Fig. 1:**
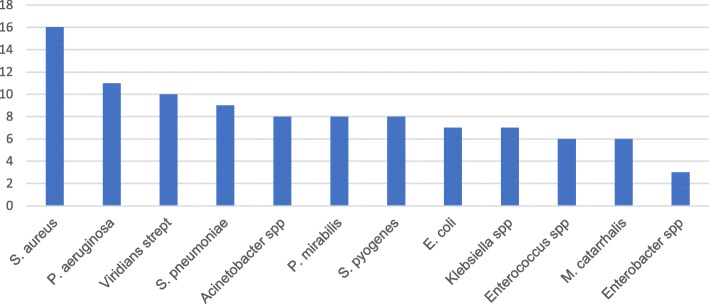
Isolated bacterial pathogens from pediatric patients with otitis media in Addis Ababa, January 2013- June 2014, Ethiopia

Ninety-five samples were culture positive making the overall prevalence of bacterial pathogen from otitis media to be 48.5%. Of these isolates, the most predominant bacterial pathogen was *S. aureus* 16 (15.8%) followed by *P. aeruginosa* 11(10.9%), Viridians streptococcus 10 (9.9%), *S. pneumoniae* 9 (8.9%) and *S. pyogenes* 8(7.9%) (Fig. 1). Six of the samples were having mixed bacterial isolate.

Previous upper respiratory tract infection and living in rural area have been observed to have statistically significant association with culture positive results (*p-value = 0.035*, *AOR = 2.011, 95% CI (1.051–3.847)*) and (*p-value = 0.003*, *AOR = 4.837, 95% CI (1.721–13.6)* respectively, thus 62.7% of children with upper respiratory tract infection history have positive culture results. But there was no statistically significant association to a single upper respiratory tract infection. The upper respiratory tract infection presented by participant children includes cold, pneumonia, tonsillitis, pharyngitis, measles, mumps and allergy (Table 2).

**Table 2:** Risk factors for bacterial otitis media in pediatric patients from Addis Ababa, Ethiopia, Jan 2013- Jun 2014 (*n* = 196)

Characteristic	Positive culture No.(%)	Negative culture No. (%)	Total prevalence No.(%)	
Residence				*X*^*2*^ *= 12.521*
Urban	74 (37.8)	96 (49)	170 (86.7)	*P-value = 0.003*
Rural	21 (10.7)	5 (2.6)	26 (13.3)	
Previous ABC usage				
Have used antibiotics	58 (29.6)	73 (37.2)	131 (66.8)	*X*^*2*^ *= 2.783* *P-value = 0.095*
Have not used antibiotics	37 (18.9)	28 (14.3)	65 (33.2)	
Upper respiratory tract infection history				
Yes	37 (18.88)	22 (11.22)	59 (30.1)	*X*^*2*^ *= 6.855* *P-value = 0.035*
No	58 (29.59)	79 (40.31)	137 (69.9)	
First ear infection				
After 1 years of age	90 (45.9)	89 (45.4)	179 (91.3)	*X*^*2*^ *= 2.707* *P-value = 0.1*
Before 1 years of age	5 (2.6)	12 (6.1)	17 (8.7)	
Total	95(48.5)	101(51.5)	*196 (100)*	

### Antimicrobial susceptibility

Most of the bacteria isolates showed > 75% susceptibility for amoxicillin-Clavulanic acid, Tobramycin, Cefotaxime, Ciprofloxacin, Cefuroxime, and Ceftazidime, while isolates were less susceptible (< 30%) for Ampicillin, Gentamycin, Penicillin G and Trimethoprim-Sulphametoxazol. *S. aureus*, Enterococcus species, Acinetobacter species and *P. aeruginosa* have been observed to have a high level of resistance to Erythromycin and Chloramphenicol (Table 3).

**Table 3:** Susceptibility pattern of bacterial isolates form pediatric patients with otitis media in Addis Ababa, Ethiopia, Jan 2013- Jun 2014 (*n* = 196)

Isolated Pathogen	Total	AMX	AMP	AUG	GEN	CHL	CIP	CLN	DOX	TOB	ERY	CEF	CTX	P	SXT	CAZ
*S. aureus*	16	4	2	13	1	6	8	10	5		4	13		1	2	
*P. aeruginosa*	11	1	1	9	2	4	7		5	10	2	9	8		2	9
Viridians strept.	10	4	2	10	1	5	10	7	7		4	8		1	1	
*S. pneumoniae*	9	4	4	8	4	6	7	5	6		5	8		3	3	
Acinetobacter spp	8	1	1	8	1	3	6	3	4	5	2	4	6	1	1	6
*P. mirabilis*	8			6	2	3	6			6		5	7		3	7
*S. pyogenes*	8	8	8	8	4	6	8	6	8		5	8		8	2	
*E. coli*	7			6	1	3	6			6		6	7		1	7
Klebsiella spp	7			4	0	0	6			5		4	7		2	7
Enterococcus spp	6	0	1	5	0	2	5	3	3	3	3	6		0	1	
M. catarrhalis*	6	2	2	6	3	2	6	2	6		3	3		2	2	
Enterobacter spp	3			3	1	2	3			3		3	3		1	3
Citrobacter spp	2			2	0	1	2			2		2	2		1	2

*AMX* Amoxicillin, *GEN* Gentamycin, *CAZ* Ceftazidime, *CHL* Chloramphenicol, *CIP* Ciprofloxacin, *CLN* Clindamycin, *AUG* Augmentin, *DOX* Doxycycline, *TOB* Tobramycin, *ERY* Erythromycin, *CEF* Cefuroxime, *CTX* Cefotaxime, *AMP* Ampicillin, *P* Penicillin G, *SXT* trimethoprim- sulfamethoxazole

*The antimicrobial susceptibility pattern of *M. catarrhalis* was based the criteria of British Society of Antimicrobial Chemotherapy [Howe and Andrews, 2012], whereas Clinical Laboratory Standards Institute [CLSI, 2013] was used for other isolates

## Discussion

Among children, peak prevalence of otitis media was observed in the age group of under five years with previous studies conducted in Ethiopia [17] and Nigeria [18–20], and the present study concentrates in this age group. This is due to the fact that younger children are more prone to otitis media related to the immaturity of their immune status, the shorter and horizontal nature of Eustachian tubes, frequent exposure to upper respiratory tract infections and malnutrition [11, 21, 22].

The overall culture positivity rate of bacterial isolates from children with otitis media in the present study was 48.5%; which is lower than other studies conducted in Nigeria 91% [23], 80.7% in Pakistan [24] and previous studies conducted in Ethiopia such as in Dessie 81.25% [7] and Gondar 87.93% [25]. This lower isolation rate could be due to high antimicrobial usage history in the area which is 66.8% which will increase the difficulty of isolating fastidious organisms like *H. influenzae, M. catarrhalis* and *S. pneumoniae*. In addition, anaerobic isolates and fungal isolates were not cultured due to lack appropriate laboratory facility.

Isolate ratio for gram positive to gram-negative bacteria in the present study was 1.27:1, which is agreement with studies from Yemen, Poland and Ethiopia, [26–28] and in contrast to previous studies that have been conducted in Ethiopia [7, 17] and Nigeria [20, 23, 29] indicating more gram-negative isolates. In the present study, 90 (93.75%) of samples were with single bacterial isolate which is similar to the other studies conducted in Finland, Ethiopia, and Nigeria [19, 27, 30, 31].

The predominant bacterial isolates in the present study were *S. aureus (15.8*%) followed by *P. aeruginosa (10.9%)*, which is similar to previous studies [23–26, 30]. Viridians streptococcus (9.9%), *S. pneumoniae* (8.9%), Acenitobacter species (7.9%), *P. mirabilias* (7.9%) and *S. pyogenes* (7.9%) include other isolated pathogens in contrast with studies [14, 29, 30, 32, 33] which isolated Enterobacteriaceae, while Jido et al. [20] indicated *P. aeruginosa* as a major isolate. The probable explanation for this variation in dominant isolates could be the climatic and geographical difference. Unlike other study which indicates *H. influenzae* prevalence to be 3.9% [34], 40% [31], 16.03% [28], 11.1% [35] and 9.7% [36] there was no *H. influenzae* isolate in this study.

Demographic factors like gender have no significant association with otitis media which agrees to some studies [31, 37], and argues to some other studies [18, 25], but some studies [38] reported that females were more affected by ear infections. This variation could be due to the cultural differences in different communities, or bias from the smaller sample size.

There have been statistically significant differences of culture positivity in those who have experienced previous upper respiratory tract and ear infection; compared to those who have no previous infection history (*p-value = 0.035*, *AOR = 2.011)*. This could be due to that fact that previous upper respiratory tract infection will disrupt the muco-ciliary mechanical defense of Eustachian tube [8, 11]. As well, there has been a significant difference of culture positivity in between children from the urban and rural area (*p-value = 0.003*, *AOR = 4.837)*. This difference could be due to the difference in personal hygiene and ear cleaning habit since bacteria responsible to ear infection are environmental organisms which can be transmitted though water and soil [39]. However, caregiver’s knowledge and habit about child care and health may have an effect since it is not assessed in the current study.

In the present study, *S. aureus* revealed > 60% level of resistance to Amoxicillin, Chloramphenicol, and Erythromycin which is in agreement with a report from Nigeria [18, 40]. But a study by Khalil et al., 2013 [24] has shown the higher sensitivity of *S. aureus* to erythromycin. Ciprofloxacin sensitivity for *S. aureus* was 50% in our study, but some other studies have shown higher sensitivity (> 80%) [32, 41, 42]. *S. aureus* isolates were resistant to gentamycin in the current study in contrast to other studies which have observed > 80% sensitivity [26, 27, 42]. *P. aeruginosa* isolates were relatively susceptible (63%) to Ciprofloxacin, > 80% susceptible to Amoxicillin -Clavulanic acid, Tobramycin, Ceftazidime and Cefuroxime comparable with another study [20, 43]. However, a study in Ethiopia by Wasihun and Zemene, 2015 [35] and Pakistan by Khalil et al. [24] have shown high resistance of *P. aeruginosa* to Amoxicillin -Clavulanic acid. But *P. aeruginosa* isolates were less susceptible to Gentamycin (18%), Chloramphenicol (36.4%), Cotrimoxazole (18%) and Erythromycin (18%) which is in consistent with other studies [19, 27, 35, 36]. This result is in contrast to studies conducted in Nigeria [20], Yemen [26] and Ethiopia [27] Sensitivity to Ciprofloxacin is in concordance with reports by Nia et al., Agrawal et al.*,* and Suhail et al.

Generally, the majority of the isolates were susceptible to Amoxicillin- Clavulanic acid, Cefuroxime, Tobramycin, Cefotaxime, and Ceftazidime have been found to be active antibiotics on the majority of isolates. The high sensitivity to those drugs has also been reported by some other authors [44]. A study conducted by Suhail et al. [42] showed the higher activity of cotrimoxazole for isolates. High level of resistance to Amoxicillin, Erythromycin, cotrimoxazole, and Chloramphenicol were observed which is comparable with results from Ethiopia [35], Nigeria [23] and Iraq [36]. However high sensitivity to Gentamicin was reported from findings in Gondar, Ethiopia [27, 45], Brazil [46] and Pakistan [47]; unlike in the present study which was 19.8%. This declined trend of sensitivity for these drugs could be due to a number of factors including inappropriate dose, sales without prescription, injudicious use [48] and developing enzymatic resistance.

### Strength and limitation of the study

The strength of the study was that all the laboratory procedures were conducted following standard operating procedures. The susceptibility test for each isolate was done three times and the average was taken. In addition, the study can help further studies to build upon on this finding as there is no published data regarding the problem in the study area. It was not possible to establish a temporal relation between the exposure and outcome variable since the study design was cross sectional study design. Plus to this, the caregiver’s knowledge and habit for child hygiene were not assessed to establish possible risk factors. The result may not be representative of pediatric patients with otitis media in Ethiopia due to small sample size.

## Conclusion

This report showed that monomicrobial infection especially by Enterobacteriaceae, Streptococcus species, *S. aureus* and *P. aeruginosa* are responsible for otitis media in < 5 children. Majority of the isolates showed antimicrobial drug resistance to commonly prescribed agents.

### Acronyms

AOM- Acute Otitis Media, ATCC- American Type Culture Collection, BSAC- British Society of Antimicrobial Chemotherapy, ENT- Ear, Nose and Throat, KIA- Kligler Iron Agar, CLSI- Clinical Laboratory Standards Institute, UK- United Kingdom, URTI- Upper Respiratory Tract Infection, USA- United States of America, WHO- World Health Organization.
